# The Effectiveness of Traditional Chinese Medicine Jinlida Granules on Glycemic Variability in Newly Diagnosed Type 2 Diabetes: A Double-Blinded, Randomized Trial

**DOI:** 10.1155/2021/6303063

**Published:** 2021-10-08

**Authors:** Jiemin Pan, Yuejie Xu, Si Chen, Yinfang Tu, Yifei Mo, Fei Gao, Jian Zhou, Cheng Hu, Weiping Jia

**Affiliations:** ^1^Department of Endocrinology and Metabolism, Shanghai Clinical Center for Diabetes, Shanghai Diabetes Institute, Shanghai Jiao Tong University Affiliated Sixth People's Hospital, Shanghai 200233, China; ^2^Shanghai Diabetes Institute, Shanghai Key Laboratory of Diabetes Mellitus, Shanghai Clinical Center for Endocrine and Metabolic Diseases, Shanghai Jiao Tong University Affiliated Sixth People's Hospital, Shanghai 200233, China

## Abstract

This study aimed to evaluate the influence of Jinlida granules on glycemic variability with or without metformin treatment in patients with newly diagnosed type 2 diabetes. This study was a 16-week, double-blinded, randomized, controlled clinical trial. The enrolled patients with newly diagnosed type 2 diabetes were randomly divided into four groups: control, Jinlida, metformin, and combination treatment groups. A retrospective continuous glucose monitoring (CGM) system was used for subcutaneous interstitial glucose monitoring for 3 days consecutively. Hemoglobin A1c (HbA1c), traditional Chinese medicine symptom score, and CGM parameters, including glucose coefficient of variation, standard deviation of blood glucose values, and time in range of glucose 3.9–10.0 mmol/L, were assessed pre-test and post-test. A total of 138 participants completed the entire procedure. Compared with the pre-test, fasting plasma glucose, 2 hour postprandial plasma glucose, HbA1c, and traditional Chinese medicine symptom score all decreased in the four groups at the end of the test, and the combination treatment group showed the most significant decrease. In terms of CGM parameters, time in range of the Jinlida and metformin groups improved after intervention compared with the baseline (Jinlida group: 78.68 ± 26.15 versus 55.47 ± 33.29; metformin group: 87.29 ± 12.21 vs. 75.44 ± 25.42; P < 0.01). Additionally, only the Jinlida group showed decreased glucose standard deviation after intervention (1.57 ± 0.61 vs. 1.96 ± 0.95; P < 0.01). Jinlida granules can improve glycemic control and glycemic variability in patients with newly diagnosed type 2 diabetes. *Clinical trial registration number*: ChiCTR-IOR-16009296.

## 1. Introduction

The national incidence of type 2 diabetes (T2D) in China is increasing annually. The estimated overall prevalence of diabetes is 10.9% among adults in China [1]. Traditional Chinese medicine (TCM) therapy for T2D has been widely used in China. TCM can provide additional benefits to patients with T2D, such as ameliorating glycemic control, improving insulin resistance and pancreatic islet function, inducing weight loss, and low incidence of adverse events [2–5]. Pharmacological studies have demonstrated that TCM can rehabilitate islet *β*-cell impairment, stimulate insulin secretion, and strengthen the utilization of glucose in peripheral tissues [6]. In the Standards of Medical Care for Type 2 Diabetes in China 2019, a section on diabetes and Chinese herbal medicine was first highlighted [7].

Jinlida is a Chinese herbal medicine approved by the China Food and Drug Administration that has been clinically used in China as an anti-diabetic agent. It is an herbal formula nourishing Pi (Spleen) and regulating body fluid of patients with diabetes, based on the TCM theory that Pi (Spleen) deficiency is involved in the pathogenesis of T2D. It is a multi-targeted hypoglycemic medication consisting of danshensu sodium salt, puerarin, salvianolic acid B, epimedin B, epimedin C, icariin, and ginsenosides Rb1, Rc, and Rb2. Evidence from pharmacological and basic researches has demonstrated its function, including protecting islet *β*-cells, anti-oxidative stress, regulating blood glucose-related hormones, and protecting vascular endothelial cells. Additionally, Jinlida can reduce insulin resistance by promoting skeletal muscle gene expression and regulating lipid metabolism, which plays a key role in the anti-diabetic effect. In recent decades, some randomized controlled clinical trials have shown the effects of Jinlida as an add-on therapy to anti-diabetic agents for the treatment of T2D, with the benefits of Jinlida being much safer and more effective than monotherapy with anti-diabetic drugs [8–14].

Besides traditional markers reflecting glycemic levels, such as hemoglobin A1c (HbA1c), glycemic dysregulation also contains markers of glycemic variability, such as glucose standard deviation (SD) and coefficient of variation of glucose levels (%CV), which can be examined and presented in detail by using a continuous glucose monitoring (CGM) system [15]. A few previous studies have reported a positive association between glycemic variability and diabetic macrovascular and microvascular complications [16].

To our knowledge, no specific study has focused on the effect of Jinlida on glycemic variability in T2D, which is correlated with diabetic vascular complications. This study aimed to evaluate the influence of Jinlida granules on glycemic variability with or without metformin treatment in newly diagnosed T2D patients. Additionally, we adopted the time in range (TIR) of glucose 3.9–10.0 mmol/L as a study outcome, because TIR is associated with the risk of diabetic microvascular complications and has been suggested as an acceptable end point for clinical trials [17–19].

## 2. Materials and Methods

### 2.1. Trial design and participants

This study was a 16-week, double-blinded, randomized, controlled clinical trial. Patients referred to the outpatient clinic at the Department of Endocrinology and Metabolism of Shanghai Jiao Tong University Affiliated Sixth People's Hospital were consecutively recruited. We enrolled patients who were newly diagnosed with T2D. The inclusion criteria were as follows: newly diagnosed medication-naive T2D; patients aged 30–70 years, man or woman; body mass index (BMI) ≥18.5kg/m^2^; 7%≤HbA1c≤10.0%; fasting plasma glucose (FPG) ≤13 mmol/L, 2-hour postprandial plasma glucose (2h-PG) ≤18 mmol/L, and patients who provided informed consent. The exclusion criteria were as follows: 1) type 1 diabetes, gestational diabetes, and miscellaneous diabetes; 2) patients with acute complications, including ketoacidosis, lactic acidosis, hyperosmolar coma, and infection in recent 1 month; 3) patients with severe heart disease, myocardial infarction, stroke, transient ischemic attacks, and peripheral arterial disease; 4) pregnant or lactating women; 5) patients with renal or hepatic dysfunction; 6) patients with systolic pressure >180 mmHg and/or diastolic pressure >110 mmHg; 7) patients with acute or chronic pancreatitis, severe cardiovascular disease, malignancy, and severe mental disease; 8) patients with alcohol or drug addiction; 9) FPG >13 mmol/L and/or 2h-PG >18 mmol/L;10) patients with allergic reactions; 11) patients with hyperthyroidism or hypothyroidism; and 12) patients with medication influencing glucose metabolism, including glucocorticoids, thyroxine and thiazide diuretics.

### 2.2. Standard protocol approvals, registrations, and patient consents

The study protocol was approved by the Ethics Committees of Shanghai Jiao Tong University Affiliated Sixth People's Hospital and was conducted in accordance with the Declaration of Helsinki (1964). Informed consent was obtained from all participants. This trial is registered at http://www.chictr.org.cn with clinical trial registration number ChiCTR-IOR-16009296.

### 2.3. Randomization, intervention, and procedure

After signing the informed consent, each patient underwent blood tests and physical examinations at the screening visit. Venous blood samples were drawn at 0800 h after a 10-h overnight fast. At least 1 week later, the patients who met the screening criteria were randomly divided into four groups according to the random encoder: group A, control group (placebo tablets + placebo granules); group B, Jinlida group (Jinlida granules + placebo tablets); group C, metformin group (metformin tablets + placebo granules); and group D, combination treatment group (Jinlida granules + metformin tablets). The patients were randomly allocated to receive medication for 16 consecutive weeks. The eligible patients underwent CGM, and a TCM symptom score (Supplement 1) was assessed before the study drugs were distributed.

All the enrolled patients were orally administered one bag of granules (Jinlida or placebo) and three tablets of pills (metformin or placebo). The dose of metformin was 500 mg three times a day after three meals from the beginning to the end of the observation period. Patients in all the groups were orally administered one bag of granules (9 g) three times daily with warm water before each meal. The HbA1c, FPG, 2h-PG, and lipid profiles were measured at 0 and 16 weeks. The HbA1c levels were measured using an analyzer (Tosoh HLC-723 G7, Yamaguchi, Japan) using high-performance liquid chromatography. The plasma glucose levels and lipid profiles were measured using an automatic biochemical analyzer (Hitachi 7600, Tokyo, Japan).

The observation period was 16 weeks with every 4-week follow-up visits. In each session, the patients were asked if there were experiencing any adverse events. All patients underwent fasting capillary glucose measurement, physical examination, and compliance with the test drug administration. BMI and blood pressure were also monitored. CGM and TCM symptom score were performed again at the 16-week follow-up visit.

Key withdrawal criteria of the study included severe hypoglycemia and serious adverse events that the investigators considered inappropriate for continuation; severe protocol deviation, including poor drug compliance, inability to continue according to protocol requirements, and unwillingness to follow the study arrangements.

### 2.4. CGM parameters

A retrospective CGM system (ipro2, Medtronic Inc., Northridge, CA, USA) was used for subcutaneous interstitial glucose monitoring for 3 days consecutively at baseline and at the end of the study. Glycemic variability was estimated using the %CV and glucose SD. CV was calculated by dividing the glucose SD by the average of the corresponding glucose readings. The CV values in this study were multiplied by 100 and expressed as %CV. TIR was defined as the percentage of time in the target glucose range of 3.9–10.0 mmol/L during a 24-hour period.

### 2.5. Outcomes

The primary outcomes were changes in HbA1c, glucose SD, and %CV compared with baseline. Secondary outcomes were changes in FPG, 2h-PG, TIR, and TCM symptom score compared with baseline. Safety analysis mainly included the incidence of self-reported hypoglycemia and gastrointestinal adverse events in the four groups.

### 2.6. Determination of sample size and statistical analyses

Since this study was a pilot study, sample size calculation was not performed, and a convenience sample size was adopted. Variables with an approximately normal distribution are presented as mean ± SD, while those with a skewed distribution are shown as medians (interquartile range). Differences in continuous variables among multiple groups were analyzed using the one-way analysis of variance (ANOVA) and post-hoc tests. Differences in the parameters before and after treatment were analyzed using the paired t-test. Skew-distributed variables were tested using the rank-sum test. Comparisons of categorical variables between groups were performed using the chi-square test. Statistical analyses were performed using the Statistical Package for the Social Sciences software (version 17.0; SPSS Inc., Chicago, IL, USA). Statistical significance was set at P < 0.05.

## 3. Results

**Figure 1:**
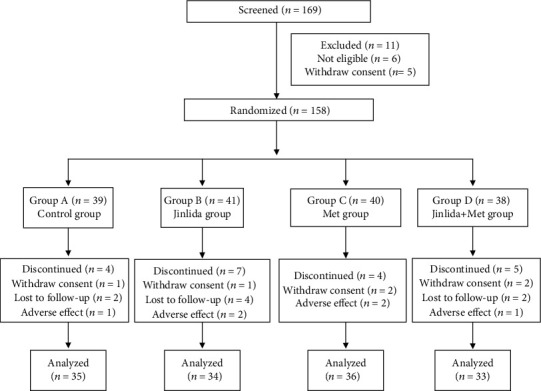
Study design and participant flow diagram.

In this study, 169 patients were screened, of whom six failed to undergo screening and five withdrew consent. In total, 158 patients were randomly assigned, of whom 20 patients discontinued, and 138 patients completed the entire procedure (Figure 1). The participants' characteristics are presented in Table 1. At baseline, there were no significant differences in the clinical parameters, including BMI, blood pressure (systolic blood pressure and diastolic blood pressure), lipid profile (total cholesterol, triglyceride, high-density lipoprotein cholesterol, and low-density lipoprotein cholesterol), metrics of glucose level (FPG, 2h-PG, and HbA1c), and TCM symptom score.

### 3.1. Glycemic level and TCM symptom score changes pre- and post-test in the four groups

**Figure 2:**
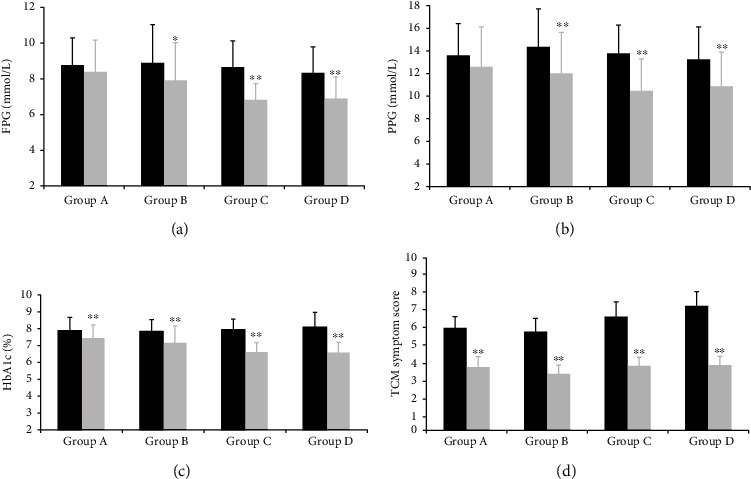
Comparison of Glycemic level and traditional Chinese medicine symptom score at pre-and post-test. (A): Comparison of fasting plasma glucose level at pre-and post-test. (B): Comparison of 2-hour postprandial plasma glucose level at pre-and post-test. (C): Comparison of HbA1c at pre-and post-test. (D): Comparison of traditional Chinese medicine symptom scores at pre-and post-test. Group A: control group; Group B: Jinlida group; Group C: metformin group and Group D: combination treatment group. FPG: fasting plasma glucose; PPG: 2 hour postprandial plasma glucose; TCM: traditional Chinese medicine. ^∗^post-test vs. pre-test, P<0.05, ^∗∗^ post-test vs. pre-test, P<0.01.

In terms of glycemic parameters, the FPG, 2h-PG, and HbA1c levels were improved compared with baseline among the four groups (Table 2) after 16-week intervention. The HbA1c levels improved with statistical significance pre-and post-tests (P < 0.01). TCM symptom score improved after invention compared with pre-intervention in the four groups. We then compared glycemic changes between the groups to explore the hypoglycemic effect of Jinlida. No significant changes were found between groups A and B and between groups C and D (Figure 2).

### 3.2. CGM measurement changes pre and post-test in the four groups

**Figure 3:**
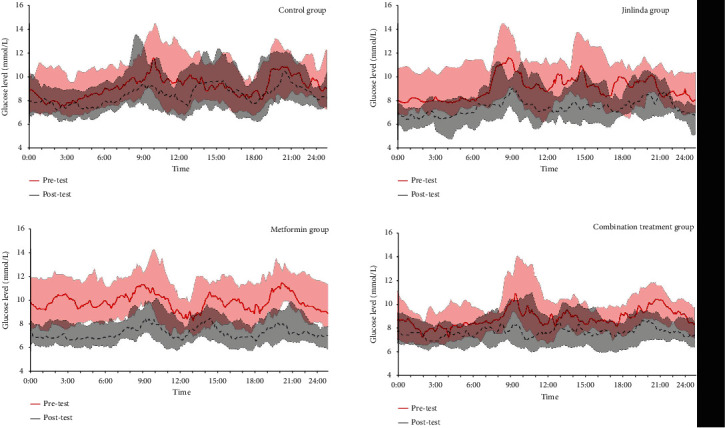
Average continuous glucose-monitoring (CGM) tracings for 72 hours at pre- and post-test. Average CGM values were calculated for each 5 min interval throughout the 3 days for each group.

The TIR of groups B and C significantly increased after the 16-week intervention (P < 0.01), while no significant changes were found in group A and group D. Further, %CV of the four groups showed no significant change pre-and post-test. However, in terms of glucose SD, group B showed a significant change after intervention compared with baseline (P < 0.01).  Figure 3 shows the average CGM measurements of all the groups.

### 3.3. Glycemic level, TCM symptom score, and CGM measurement changes in multiple groups

The ANOVA analysis showed significant differences in the FPG, 2h-PG, and HbA1c levels in multiple groups (F = 4.972, 2.763, 10.703, P < 0.05). To further explore the effects of Jinlida on glycemic level, TCM symptom score, and CGM measurements, we compared the pre-and post-test changes between groups A and B, and groups C and D with a post-hoc test. However, no significant differences were found in the FPG, 2h-PG and HbA1c levels. In addition, there was no significant difference in the TCM symptom score between the groups (group A vs. group B, group C vs. group D) (Figure 2).

**Figure 4:**
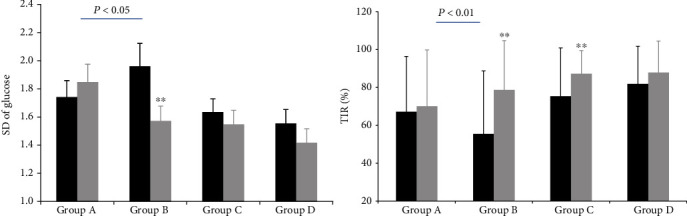
Comparison of standard deviation of glucose and time in range between pre-and post-test. SD: standard deviation; TIR: time in range. ^∗^post-test vs. pre-test, P<0.05, ^∗∗^ post-test vs. pre-test, P<0.01. The improvement of SD showed significant difference between control group and Jinlida group (P<0.05). The change of TIR was found significant difference between control group and Jinlida group (P<0.01).

On the contrary, the improvement of glycemic variability assessed by glucose SD showed significant difference between the Jinlida and control groups (glucose SD, -0.39 vs. 0.11 P < 0.05). However, compared with the metformin group, no significant improvement in glucose SD post-study was found in the combination treatment group (glucose SD, -0.18 vs. -0.09, P > 0.05). Additionally, compared with the control group, the Jinlida group showed a more pronounced improvement in TIR after treatment (23.21% vs. 2.24%, P < 0.01). Since there was a significant difference in the metrics of TIR among the four groups at baseline, we compared the percentage increase from baseline in multiple groups after 16 weeks of intervention. No significant difference was found in multiple groups with the ANOVA analysis (F = 0.796, P > 0.05), or between groups A and B and groups C and group D with post-hoc test (P > 0.05) (Figure 4).

### 3.4. Adverse events

During the study period, adverse events (except for hypoglycemia) occurred two times in group A, seven times in group B, 13 times in group C, and nine times in group D. Four cases of gastrointestinal discomfort in group C and three cases in group D were related to the study drugs, while the other adverse events were not related to the study drugs. Hypoglycemia was observed in one patient in group B and in one patient in group D throughout the study, and none of them experienced severe hypoglycemia.

## 4. Discussion

TCM has focused on the treatment of diabetes for thousands of years. The “whole view” and “multi-target” approaches of TCM provide unique advantages in controlling complex metabolic diseases, such as diabetes. It usually focuses on individualized treatments that are based on the differentiation of syndromes, control of balance, and various routes of administration. In recent years, large-scale clinical trials have confirmed that TCM has progressed in controlling blood glucose levels [20–24].

Jinlida is an herbal formula originating from the Chinese cognition of the diabetes onset theory, “Pi (Spleen) dysfunction,” which was first described in Lingshu, a famous ancient Chinese medical book. Jinlida was made to nourish Pi and regulate body fluid, and consists of ginseng (Renshen), pale white atractylodes rhizome (Cangbaizhu), Poria cocos (Fuling), the root of kudzu vine (Gegen), and radix polygonati officinalis (Yuzhu). Accordingly, the combined herbs tonify the Pi, facilitate the circulation of Qi (energy), and nourish yin [13].

In this study, it was found that after 16 weeks of treatment, the Jinlida, metformin, and combination treatment groups all showed a significant reduction in the HbA1c levels, but there was no significant difference in the magnitude of HbA1c reduction among the three groups. These results are consistent with those of previous studies. Several clinical studies have been conducted in China to assess the efficacy of Jinlida for T2D treatment. Tian et al. conducted a 12-week multicenter double-blind controlled trial to evaluate whether Jinlida can enhance glycemic control in T2D patients with metformin treatment. It was found that HbA1c was reduced by 0.53% with Jinlida in the subgroup with baseline HbA1c ≤ 7.5% [12]. This study showed similar results: HbA1c was reduced by 0.68% with Jinlida monotherapy. Compared with metformin monotherapy, combination treatment with Jinlida reduced HbA1c by 0.18%. The mechanism of the hypoglycemic effect of Jinlida could be related to its protection of pancreatic secretory function through adenosine monophosphate-activated protein kinase activation [6].

To our knowledge, this is the first study to evaluate TIR in all patients with T2D using CGM. TIR is associated with the risk of diabetic microvascular complications and should be accepted as a reasonable glycemic metric to assess glycemic control [25]. In general, a TIR of 3.9–10.0 mmol/L is a useful parameter to evaluate the treatment regimen [17]. Vigersky et al. found a good correlation between TIR and HbA1c based on 18 randomized controlled trials, including patients with type 1 diabetes and T2D. Further, 70% and 80% of TIR were estimated to be equivalent to an HbA1c level of 6.7% and 5.9%, respectively [26]. We found that Jinlida monotherapy significantly increased TIR by 23% compared to baseline TIR. However, after adjusting for baseline TIR, we did not find an obvious change in the pre-and post-test TIR in multiple groups. The Standards of Care for T2D in China (2019) first included the section on Diabetes and TCM and recommended the addition of Jinlida granules orally in T2D with deficiency of both Qi and Yin and poor efficacy of metformin monotherapy [7]. In this study, we examined the effect of Jinlida granules on TIR improvement and found that Jinlida granules could be an alternative to metformin in newly onset T2D with mild glucose elevation. Additionally, no obvious adverse events were reported, indicating that this herbal medication is safe for clinical use.

Glycemic variability is another part of glucose dysregulation, which also contributes to diabetic vascular complications. Glycemic variability is usually assessed by the parameters of glucose SD and %CV, adopting CGM [16, 27]. In our study, we found that Jinlida granules could reduce glucose SD significantly compared to the control group. Accordingly, the Jinlida granules may improve glycemic variability. In previous research, treatment with Jinlida reduced hepatic oxidative stress, which may lead to the improvement of glycemic variability [2].

This study had some limitations. This was a small sample study with approximately 40 patients in each group. Before performing CGM, all patients did not receive standard dietary guidance. These reasons may lead to the significantly increased TIR levels in group D at baseline and no significant change in TIR in group D after treatment. To further determine the effect of Jinlida granules on glycemic control and glycemic variability, a large sample study is necessary. We only found that Jinlida granules can improve glucose SD significantly compared with the control group (P < 0.01), and significant changes in the HbA1c, TIR, and glucose SD levels were not found between the metformin and combination treatment groups. This may be because of the sample size and study period. The baseline difference in TIR may lead to statistical bias, and the sample size should be further expanded. Further study is needed to investigate differences in metabolomics among all the groups.

## 5. Conclusions

Jinlida granules can improve glycemic control and glycemic variability in patients with newly diagnosed T2D.

## Figures and Tables

**Table 1:** Baseline characteristics of the subjects in the study.

Variable	Group A	Group B	Group C	Group D	*F* value	*P* value
Samples (n)	35	34	36	33		
Age (years)	56.53 ± 9.18	51.59 ± 10.31	56.00 ± 9.47	54.57 ± 10.54	1.733	0.163
Sex (male/female)	23/12	25/9	24/12	22/11		0.892
BMI (kg/m2)	25.69 ± 3.24	25.40 ± 3.97	26.60 ± 3.42	25.74 ± 2.73	0.830	0.480
FPG (mmol/L)	8.77±1.53	8.89±2.15	8.65±1.48	8.33±1.46	0.667	0.574
2h-PG (mmol/L)	13.60±2.83	14.35±3.36	13.79±2.48	13.26±2.86	8.834	0.478
HbA1c (%)	7.88 ± 0.78	7.83 ± 0.69	7.94 ±0.63	8.09 ±0.88	0.731	0.535
FINS (mU/L)	13.73±7.58	14.42±10.31	14.65±7.92	12.50±6.99	0.455	0.714
2h-INS(mU/L)	63.45±47.01	69.40±58.95	60.48±31.76	46.20±33.54	1.663	0.178
SBP(mmHg)	136.06±13.32	136.38±15.89	137.28±18.39	131.30±15.56	0.958	0.415
DBP(mmHg)	83.06±7.64	84.15±8.99	84.61±12.41	80.42±9.65	1.220	0.305
Triglyceride (mmol/L)	1.66 ± 0.80	2.17 ± 1.39	1.81 ± 0.91	2.15 ±2.12	1.136	0.337
Total cholesterol (mmol/L)	4.91 ± 1.03	5.17 ± 0.91	5.10± 1.00	5.32±1.03	0.966	0.411
LDL-C (mmol/L)	3.18 ± 1.01	2.97 ± 0.86	3.19 ± 0.81	3.30 ±0.97	0.742	0.529
HDL-C (mmol/L)	1.13 ± 0.23	1.15± 0.26	1.16 ± 0.28	1.20 ±0.30	0.384	0.765
TCM symptom scale	7.14 ± 4.74	6.88 ±5.38	7.94 ±6.13	8.67±5.67	0.729	0.536
TIR (%)	67.15±29.29	55.47±33.29	75.44±25.42	81.87±19.89	5.661	0.001
SD	1.74±0.69	1.96±0.95	1.63±0.57	1.55±0.56	2.079	0.106
CV (%)	18.87±5.83	20.28±8.60	18.74±5.15	18.94±6.27	0.410	0.746

Group A, control group; Group B, Jinlida group; Group C, metformin group and Group D, combination with Jinlida and metformin group. BMI, body mass index; FPG, fasting plasma glucose; 2h-PG, 2-hour postprandial glucose; HbA1c, hemoglobin A1c; FINS, fasting insulin; 2h-INS, 2 hour insulin; SBP, systolic blood pressure; DBP, diastolic blood pressure; LDL-C, low density lipoprotein cholesterol; HDL-C, high density lipoprotein cholesterol; TCM, traditional Chinese medicine; TIR, time in range; SD, standard difference; CV, coefficient of variation.

**Table 2:** Glycemic changes pre- and postinvention.

Variable	Group A *n* = 35		Group B *n* = 34		Group C *n* = 36		Group D *n* = 33	
	Pretest	Posttest	Pretest	Posttest	Pretest	Posttest	Pretest	Posttest
FPG (mmol/L)	8.77 ± 1.53	8.40 ± 1.77	8.89 ± 2.15	7.91 ± 2.11∗	8.65 ± 1.48	6.82 ± 0.90∗∗	8.33 ± 1.46	6.90 ± 1.21∗∗
2 h-PG (mmol/L)	13.60 ± 2.83	12.59 ± 3.52	14.35 ± 3.36	12.02 ± 3.62∗∗	13.79 ± 2.48	10.46 ± 2.84∗∗	13.26 ± 2.86	10.87 ± 3.02∗∗
HbA1c (%)	7.88 ± 0.78	7.43 ± 0.79∗∗	7.83 ± 0.69	7.15 ± 1.01∗∗	7.94 ± 0.63	6.61 ± 0.57∗∗	8.09 ± 0.88	6.58 ± 0.61∗∗
TCM symptom scale	7.14 ± 4.74	4.40 ± 4.30∗∗	6.88 ± 5.38	3.94 ± 3.50∗∗	7.94 ± 6.13	4.50 ± 3.46∗∗	8.67 ± 5.67	4.55 ± 3.44∗∗
TIR (%)	67.15 ± 29.29	70.06 ± 29.77	55.47 ± 33.29	78.68 ± 26.15∗∗	75.44 ± 25.42	87.29 ± 12.21∗∗	81.87 ± 19.89	87.87 ± 16.69
SD	1.74 ± 0.69	1.85 ± 0.75	1.96 ± 0.95	1.57 ± 0.61∗∗	1.63 ± 0.57	1.55 ± 0.59	1.55 ± 0.56	1.42 ± 0.55
CV (%)	18.87 ± 5.83	20.83 ± 6.65	20.28 ± 8.60	18.62 ± 5.60	18.74 ± 5.15	19.73 ± 5.63	18.94 ± 6.27	18.42 ± 5.73

## Data Availability

The original data sets of this study are available on request to the corresponding author.

## Supplementary Materials

Supplementary Materials Traditional Chinese Medicine Symptom Grading and Quantitative Standard
