# Supplementary material for: The Effectiveness of Traditional Chinese Medicine Jinlida Granules on Glycemic Variability in Newly Diagnosed Type 2 Diabetes: A Double-Blinded, Randomized Trial
Source: J Diabetes Res. 2021 Oct 8;2021:6303063. doi: 10.1155/2021/6303063 (PMC8519714; doi:10.1155/2021/6303063)
Supplement: Supplementary Materials — Traditional Chinese Medicine Symptom Grading and Quantitative Standard [file 6303063.f1.docx]

Traditional Chinese Medicine Symptom Grading and Quantitative Standard

Based on the《Guiding Principles for Clinical Research on New Traditional Chinese Medicines》 published by the Ministry of Health in 1993 and 《Guiding Principles for Clinical Research on New Traditional Chinese Medicines for Diabetes》 published by China Medical Science and Technology Press in 2002

1) Thirsty and polydipsia:

Asymptomatic 0 points

Drinking slightly more than ever 2 points

Drinking more than half as much water as ever 　 4 points

Drinking water more than doubled 6 points

2) Lassitude and fatigue:

Asymptomatic 0 points

Easy to tired, can insist on physical labor 2 points

Mental fatigue, reluctant support for daily labor 4 points

Weak limbs, unable to support daily labor 6 points

3) Overeating and sense of hunger:

Asymptomatic 0 points

Obvious sense of hunger 1 point

Unbearable sense of hunger before meals 2 points

Unbearable sense of hunger, Susceptibility to hypoglycemia 3 points

4) Excessive urine:

Asymptomatic 0 points

A slight increase in urine volume, less than 2 liters per day 1 point

Increased urine volume, less than 2-3 liters per day 2 points

Significantly increased urine volume, less than 3 liters per day 3 points

5) Pharyngeal and mouth drying

Asymptomatic 0 points

Slightly pharyngeal and mouth drying, can be relieved by a little drinking 1 point

Pharyngeal and mouth drying can be relieved by drinking water 2 points

Pharyngeal and mouth drying are unbearable, hard to be relieved by drinking 3 points

6) Spontaneous sweating:

Asymptomatic 0 points

Sweat slightly after the activity, wet clothes slightly 1 point

Inactive skin moist, significantly increased after slight movement 2 points

Inactive sweating, sweat like water stains after movement 3 points

7) Night sweats:

Asymptomatic 0 points

Sweat on the head and occasionally appear 1 point

Moist chest and back, recurring 2 points

The whole body is damp like washing, often appear 3 points

8) Burning sensation of five centres:

Asymptomatic 0 points

Hot hands, feet, and heart at night, occasionally upset 1 point

Hot hands and feet, sometimes upset 2 points

Burning hands, feet and heart, Desire for cold things in hands, upset all day long 3 points

9) Constipation:

Asymptomatic 0 points

Dry stool, once a day 1 point

Dry and hard stool, once every 2-3 days 2 points

Difficulty with dry and hard stool, once more than four days 3 points
